# Uncovering the evolutionary history of neo-XY sex chromosomes in the grasshopper *Ronderosia bergii* (Orthoptera, Melanoplinae) through satellite DNA analysis

**DOI:** 10.1186/s12862-017-1113-x

**Published:** 2018-01-08

**Authors:** Octavio M. Palacios-Gimenez, Diogo Milani, Bernardo Lemos, Elio R. Castillo, Dardo A. Martí, Erica Ramos, Cesar Martins, Diogo C. Cabral-de-Mello

**Affiliations:** 1 Departamento de Biologia, UNESP - Univ Estadual Paulista, Instituto de Biociências/IB, Rio Claro, São Paulo 13506-900 Brazil; 2 Program in Molecular and Integrative Physiological Sciences, Department of Environmental Health, Harvard University T. H. Chan School of Public Health, Boston, Massachusetts 02115 USA; 3 IBS - UNaM – CONICET, Posadas, Misiones Argentina; 4 Departamento de Morfologia, UNESP – Univ Estadual Paulista, Instituto de Biociências/IB, Botucatu, São Paulo Brazil

**Keywords:** Chromosomal rearrangements, Evolution, FISH, Satellite DNA, Sex chromosome

## Abstract

**Background:**

Neo-sex chromosome systems arose independently multiple times in evolution, presenting the remarkable characteristic of repetitive DNAs accumulation. Among grasshoppers, occurrence of neo-XY was repeatedly noticed in Melanoplinae. Here we analyzed the most abundant tandem repeats of *R. bergii* (2n = 22, neo-XY♂) using deep Illumina sequencing and graph-based clustering in order to address the neo-sex chromosomes evolution.

**Results:**

The analyses revealed ten families of satDNAs comprising about ~1% of the male genome, which occupied mainly C-positive regions of autosomes. Regarding the sex chromosomes, satDNAs were recorded within centromeric or interstitial regions of the neo-X chromosome and four satDNAs occurred in the neo-Y, two of them being exclusive (Rber248 and Rber299). Using a combination of probes we uncovered five well-defined cytological variants for neo-Y, originated by multiple paracentric inversions and satDNA amplification, besides fragmented neo-Y. These neo-Y variants were distinct in frequency between embryos and adult males.

**Conclusions:**

The genomic data together with cytogenetic mapping enabled us to better understand the neo-sex chromosome dynamics in grasshoppers, reinforcing differentiation of neo-X and neo-Y and revealing the occurrence of multiple additional rearrangements involved in the neo-Y evolution of *R. bergii*. We discussed the possible causes that led to differences in frequency for the neo-Y variants between embryos and adults. Finally we hypothesize about the role of DNA satellites in *R. bergii* as well as putative historical events involved in the evolution of the *R. bergii* neo-XY.

**Electronic supplementary material:**

The online version of this article (10.1186/s12862-017-1113-x) contains supplementary material, which is available to authorized users.

## Background

Evolutionary evidence indicates that sex chromosomes have evolved independently from ordinary autosomal pairs (the proto-XY or the proto-WZ pairs) comprising one of the most dynamic chromosomal elements in the genome [1–3]. The sex chromosome systems are diverse with, for example, the XY system in mammals, the WZ system in Lepidoptera, birds and some reptiles. Moreover, the occurrence of multiple sex chromosomes (multiple Xs and Ys or Zs and Ws) is also a common phenomenon [4–8]. Common hallmarks of sex chromosome evolution include the partial or complete loss of recombination between them; abundant gene inactivation or loss (i.e., genetic degeneration); progressive accumulation/expansion of repetitive DNAs; and heterochromatinization of the Y or W chromosomes [6, 7, 9–14].

In Orthoptera the X0♂/XX♀ sex chromosome system is a conserved pattern found in most species. This modal system results from the loss of the Y chromosome in an ancestral XY♂/XX♀ species [15, 16]. However, derived variants, like neo-XY♂/neo-XX♀, neo-X_1_X_2_Y♂/neo-X_1_X_1_X_2_X_2_♀ or even X_1_X_2_0♂/X_1_X_1_X_2_X_2_♀ evolved several times due to chromosomal rearrangements involving autosomes and sex chromosomes [15–21]. In animals, it is particularly evident that chromosomal rearrangements trigger recombination suppression because of the lack of homologous sequences in regions participating in the rearrangement [22–25]. The establishment of inversions, for example, is typically associated with recombination suppression and has been detected in the Y chromosome of the grasshopper *Ronderosia bergii* [20], mammals [23], the three-spine stickleback fish [26], and the plant *Silene latifolia* (Nicolas et al. [11]).

*Ronderosia bergii* is a Melanoplinae grasshopper with a 2n = 22, neo-XY♂ karyotype. The neo-X is metacentric and the neo-Y is acrocentric, with a larger small arm in comparison to autosomes, and rich in heterochromatin; they are the result of an X-A centric fusion followed by a large pericentric inversion involving more than 90% of the neo-Y length. The inversion restricted the segment of contact between the chromosomes to the distal regions during meiosis [20, 27, 28]. There is no published estimate about the origin time of *Ronderosia* genus to accurately estimate the age of the neo-XY system in *R. bergii*. However, the genus is endemic to South America [29] and members of the Melanoploid lineage have been present for at least 46 mya in this region [30]. Classical cytogenetic and molecular analyses suggest that the *R. bergii* neo-Y has acquired derived features as well as retained ancestral features after its origin. For instance, the neo-Y chromosome accumulated repetitive DNA sequences while retaining the post-translational histone modifications of autosomes [20].

The heterochromatic nature of the *R. bergii* neo-Y chromosome suggests the accumulation of repetitive DNAs (e.g., satellite DNAs, satDNAs), as detected in the neo-Y of the plants *Silene latifolia* [31] and *Rumex acetosa* [32], the cervid *Muntiacus muntjac* [33, 34], among others. Here we performed a characterization of the most abundant satDNAs in the *R. bergii* genome. The analyses are mostly focused on the neo-XY sex chromosomes, and the expectation that information about sex chromosome origin and evolution could be obtained from satDNAs. We found that satDNAs predominate at centromeric heterochromatin of autosomes and neo-X chromosome, and throughout the neo-Y. Interestingly, FISH chromosomal localization of satDNAs in both embryonic neuroblasts and adult male metaphases revealed five neo-Y variants, explained by the occurrence of paracentric inversions involving two of the satDNAs identified here. In addition, amplification of two other satDNAs significantly increased the size of the neo-Y. This data brings new information about neo-XY sex chromosome evolution in *R. bergii*, revealing that the evolutionary history of these chromosomes is much more complex than previously thought.

## Methods

### Sampling, chromosome obtaining and DNA extraction

Adult animals (males and females) of *Ronderosia bergii* were collected in the Parque Estadual Edmundo Navarro de Andrade (Rio Claro, SP, Brazil) between May/2013 and March/2015 under authorization of COTEC (process number 341/2013). Testes were removed and fixed in Carnoy’s solution (3:1, 100% ethanol: absolute Acetic Acid). For embryo obtaining females were kept in captivity until oviposition. Mitotic chromosomes were obtained following the protocol proposed by Webb et al. [35]. The phenol/chloroform-based procedure described in Sambrook and Russel [36] was used to obtain genomic DNA from males and females. For karyotypic analyses it was used the conventional staining with 5% Giemsa. White’s [15] terminology was used to recognize the arms of the neo-X chromosome at meiosis: the arm from the original X chromosome was referred to as XL and the arm that shares homology with the neo-Y was referred to as XR.

### Illumina sequencing and graph-based clustering

Paired-ends libraries (2 × 300) were prepared as recommended by Illumina (Illumina Inc., San Diego, CA, USA), using Nextera DNA kit and genomic DNA isolated from a single male. Paired-end reads were preprocessed to check the quality of sequenced nucleotides with FASTQC [37] and quality trimmed using the FASTX-Toolkit suit [38]. The trimmed paired-end reads were joined using the “fastq-join” software of the FASTX-Toolkit suit [38] with default options. To search for satDNAs in the *R. bergii* genome, 4,702,802 joined reads were used in the RepeatExplorer pipeline [39, 40]. We then searched for clusters showing higher graph density, which is a typical feature of satDNAs families analysis [39, 40] due to tandem repeats characteristic of the sequence.

### satDNAs isolation and sequence analysis

The contigs from clusters displaying high graph density were submitted to Tandem Repeats Finder (TRF) algorithm [41]; TRF enabled the identification of DNA sequences that maximized the alignment scores between the different tandemly repeated monomers. We used 2, 3, 5 parameters for match, mismatch and indels, respectively in TRF analysis, and the score of 50 for minimum alignment was considered for reporting. To confirm the tandem organization and to identify monomers of the same family, we used the dotplot graphic alignment tool implemented in Dotlet [42]. To identify a representative copy of a given satDNA family, the monomers with maximum length was taken. To check similarity with published sequences, monomers were used as the query in BLAST and Repbase searches using the National Center for Biotechnology Information (NCBI) [43] and RepeatMasking tools [44]. In addition, these canonical monomers were BLASTed against the satellitome of *Locusta migratoria* [45] and *Eumigus monticola* [46]. satDNAs copies were aligned using Muscle [47] implemented in MEGA5 [48]. Nucleotide divergence (*p* divergence) and A + T content were estimated in MEGA5.

The presence of satDNAs families in both male and female were verified by polymerase chain reactions (PCR). The primers were designed based on consensus sequence of each satDNA family using the Primer3 software [49] or manually. PCR mix reactions contained 10× PCR Rxn Buffer, 0.2 mM MgCl_2_, 0.16 mM dNTPs, 2 mM of each primer, 1 U of *Taq* Platinum DNA Polymerase (Invitrogen, San Diego, CA, USA) and 50-100 ng/μl of template DNA. The PCR conditions were as follows: initial denaturation at 94 °C for 5 min and 30 cycles at 94 °C (30 s), 55 °C (30 s), and 72 °C (80 s), plus a final extension at 72 °C for 5 min. Monomeric bands were isolated and purified from agarose gel 1% using the Zymoclean™ Gel DNA Recovery Kit (Zymo Research Corp., The Epigenetics Company, USA) according to the recommendations of the fabricant. Afterwards, the monomers were used as template for PCR reamplification.

The purified PCR products were sequenced in both directions (Macrogen Inc., South Korea) in order to determine the isolation of the sequences of interest. The sequenced products were compared to the consensus sequences obtained by genome analysis. The monomer consensus sequences belonging to each of the ten satDNAs families obtained from genomic analysis were deposited into the NCBI database under the following accession numbers: MF765804-MF765813.

### Probes and Fluorescence *In situ* Hybridization, measurement of sex chromosomes and distance between signals

The PCR products for each satDNA family with more than 50 bp were labeled by nick translation using biotin-14-dATP (Invitrogen) or digoxigenin-11-dUTP (Roche, Mannheim, Germany). SatDNAs with less than 50 bp were labeled directly at the 5′ end with biotin-14 dATP (Sigma-Aldrich, St Louis, MO, USA) during their synthesis. For single or two-color FISH the protocols proposed Pinkel et al. [50] with modifications [51] were followed using mitotic chromosome preparations. Probes labeled with digoxigenin-11-dUTP were detected using anti-digoxigenin rhodamine (Roche), while the probes labeled with biotin-14-dATP were detected using Streptavidin Alexa Fluor 488-conjugated (Invitrogen). The preparations were mounted in VECTASHIELD (Vector, Burlingame, CA, USA) with chromosomes counterstained using 4′,6-diamidine-2′-phenylindole (DAPI).

The sex chromosome sizes and the distance between some FISH signals present in neo-Y chromosome were measured using the software ImageTool version 3.0 (developed at the University of Texas Health Science Center at San Antonio, Texas and available from the Internet by anonymous FTP from maxrad6.uthscsa.edu).

## Results

### SatDNAs identification and sequence analysis

Clustering analysis with the RepeatExplorer pipeline [39, 40] produced 1,030,168 clusters (containing 91,4% of the raw reads) with differences in size and genome composition. It included satDNAs and other non-characterized repetitive elements. A low copy number fraction of the genome is represented by singletons containing 8.6% of reads. For satDNAs searching the most abundant clusters (551 clusters) representing repetitive elements were examined.

The analysis of clusters generated by RepeatExplorer and using TRF algorithm, besides dotplots confirmed that 10 of the 551 clusters were resolved to be satDNAs sequences, with repeat units ranging from 5 to 285 bp long. Among them, six satDNAs sequences presented >50 bp length and were experimentally validated through PCR. The analyses revealed a ladder pattern in the gel electrophoresis that is characteristic for this kind of repetitive sequence. For the remaining four repeat families, with monomers <50 bp, we were not able to confirm them due to the very limited sequence window for PCR primer design and amplification. Features for satDNAs families are detailed in the Table 1. According to RepeatExplorer output all 10 satDNAs families together represented about 1.071% of the male genome, with the most abundant representing 0.551% and the less abundant 0.011% of the genome. Within satDNAs families the nucleotide divergence ranged from 6.2 to 17.9%. A + T content varied from 39.1 to 64.8% (Table 1). NCBI BLAST, internal BLAST, and Repbase searches performed using the consensus monomer sequence belonging to each repeat family as query did not reveal significant similarity with described sequences. The monomer consensus sequences of each satDNA family are shown in the Additional file 1. The satellites alignments are available upon request to the author.

**Table 1:** Main features of the satDNAs families isolated from the *R.bergii* genome. The FISH signals on the neo-Y chromosome corresponding to the most common pattern seen at mitosis

Repeat family	Monomer length (bp)	AT%	Genome proportion%	Nucleotide divergence % (±se)	Reads/Contigs	Chromosomal position detected by FISH											
						1	2	3	4	5	6	7	8	9	10	neo-X	neo-Y
Rber1	52	63.6	0.551	16.4 (±1)	34.9	c	c	c	c	–	–	–	–	–	c	c	p
Rber59	22	39.1	0.152	10.8 (±2.4)	475	c	c	c	c	c	–	–	c	–	–	c	
Rber61	11	63.6	0.146	15.2 (±3.2)	143.18	c,p	c	c	c	c	c	c,p	–	–	c	c	–
Rber158	177	52.9	0.064	16.4 (±1.6)	110.7	–	–	–	–	–	–	–	–	c, p	–	i	3i,p
Rber185	22	56.5	0.053	8.7 (±2.5)	832	–	–	–	c	–	i	i	–	–	–	–	–
Rber248	165	59.4	0.035	13.6 (±2.2)	86.05	–	–	–	–	–	–	–	–	–	–	–	p
Rber299	285	55.1	0.028	6.2 (±1.1)	323	–	–	–	–	–	–	–	–	–	–	–	p
Rber370	16	56.2	0.019	17.9 (±1.7)	15.78	c	c	c	c	c		c	–	–	c	c	–
Rber491	121	64.8	0.012	12.6 (±1.5)	25.82	c	c,d	c,d	c,d	c,i,d	c,d	c,d	c	–	c	c	–
Rber520	5	60	0.011	16.1 (±8.3)	64.12	c	c	c	c	c	–	–	c	–	–	c	–

*±se* standard error, *c* centromeric, *i* interstitial, *d* distal, *p* proximal

### Chromosomal localization of satDNAs

**Fig. 1:**
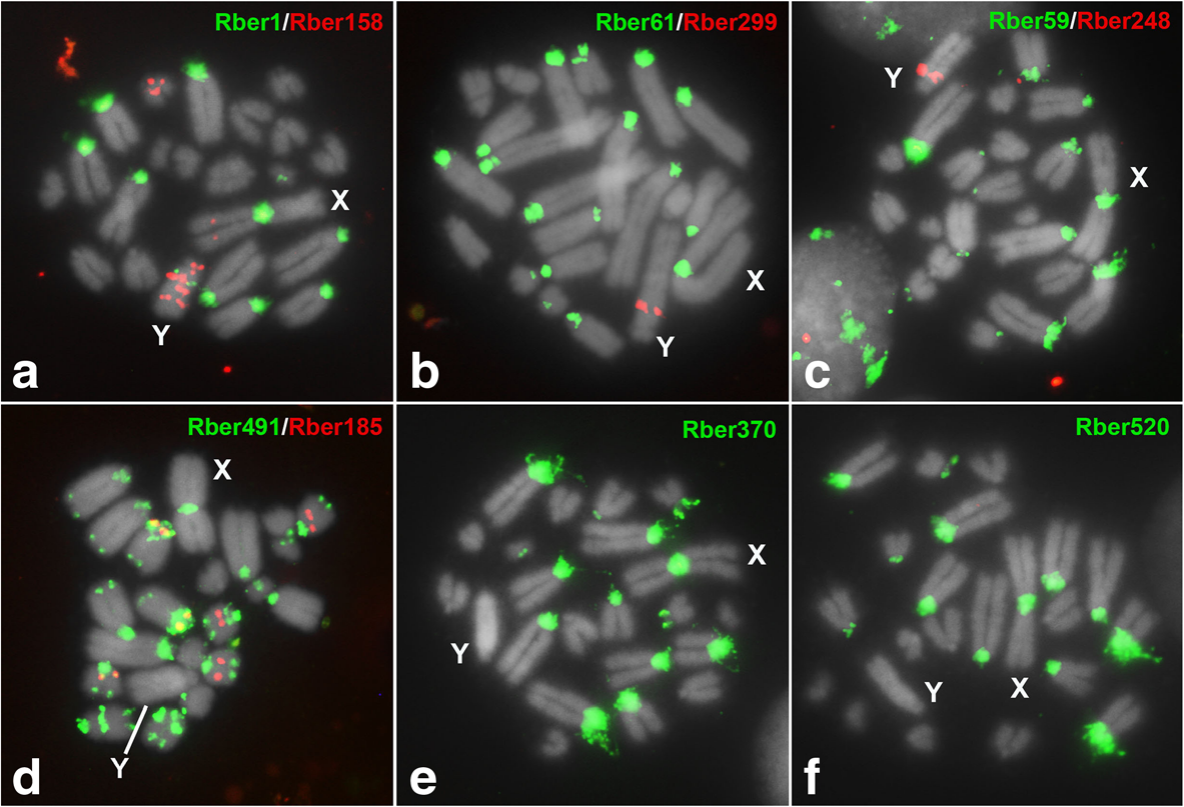
Fluorescent in situ hybridization in male embryo mitotic metaphase for the ten satDNAs recovered from *R. bergii* genome (**a**-**f**). Note the divergent pattern of location depending of the repeat (**a**-**f**) and the exclusive occurrence of Rber248 (**c**) and Rber299 (**b**) in the neo-Y chromosome. The sex chromosomes are directly indicated in each panel, as the specific satDNA family mapped

**Fig. 2:**
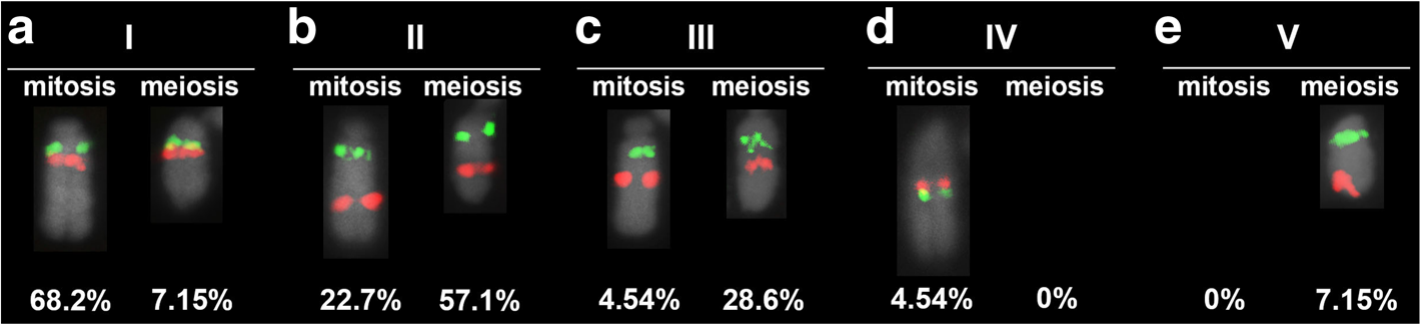
Neo-Y five chromosomes variants revealed by physical mapping of the two satDNAs exclusive of neo-Y chromosome, i.e. Rber248 (green) and Rber299 (red). Note in (**a**-**c**, **e**) that the Rber248 is maintained in the same position while the Rber299 is involved in the paracentric inversion, that involved distinct sizes of the neo-Y. In **d** note the enlargement of the neo-Y and that the Rber248 and Rber299 are maintained near each other, but in inverted order in comparison to the other neo-Y variants. The percentages indicate the proportion of individuals harboring the distinct variants of neo-Y in embryos (mitosis) and adults (meiosis)

The ten satDNAs we identified were represented on both autosomal and sex chromosomes. As expected, satDNAs were localized primarily in segments of heterochromatin (i.e. pericentromeric areas), although non-heterochromatic segments were also labeled, including interstitial, proximal or terminal regions. Most of the 10 satDNAs occurred in several chromosomes but none was present in all chromosomes (Fig. 1, Table 1). Some of the satDNAs were localized on some autosomes (Rber1, Rber59, Rber61, Rber370, Rber491, Rber520), while other were restricted to few chromosome pairs, like Rber158, Rber185 (Fig. 1, Table 1). Two satDNAs were exclusively visualized through FISH in the neo-Y chromosome [Rber248 (Fig. 1c) and Rber299 (Fig. 1b)], with proximal segments, but not co-localized (Fig. 2). These two satDNAs were involved in paracentric inversions (see below). Besides these two satDNAs the neo-Y also harbored Rber1 and Rber158. Rber1 was mapped to a small proximal band while Rber158 was localized in three different segments in the long arm and one segment in the short arm of the neo-Y chromosome (Fig. 1a). Finally, seven satDNAs were present in the neo-X chromosome, six of them in the centromeric region and one (Rber158) in the interstitial position (Fig. 1, Table 1).

### Variable neo-Y chromosomes with different satDNAs distribution

**Fig. 3:**
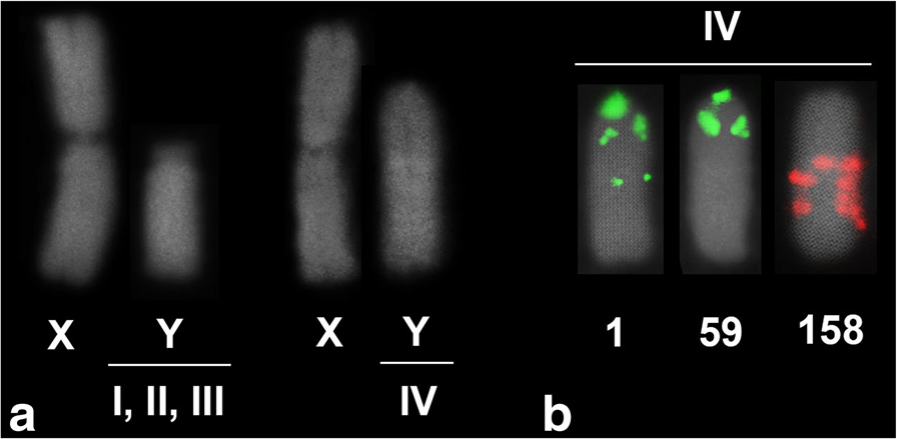
**a** Selected neo-XY chromosomes showing the differences in size for neo-X and neo-Y for the four variants observed in mitosis. **b** FISH using three distinct satDNAs families in the neo-Y variant IV

Intriguingly, we observed inter-individual variation in neo-Y chromosomes size and variable localization of the satDNAs that are exclusively placed in the neo-Y (Rber248 and Rber299). Regarding the size of the neo-Y two variants were observed in distinct males, one in which the neo-Y is almost half size of the neo-X chromosome and another one in which the neo-Y is about 0.78 of the neo-X chromosome observed in one individual (Fig. 3a; Additional file 2).

Concerning the location of Rber248 and Rber299, we studied 15 adult males and 22 embryos. The study of 22 embryos sampled randomly from distinct ooteca revealed four inter-individual distinct variants, named as I, II, III and IV. Pattern I was the most common; it occurred in 68% of the embryos (15 embryos) and showed that Rber248 was proximal to centromere while Rber299 was located just below this marker (Fig. 2a). In pattern II, Rber248 was proximal to the centromere while the Rber299 was located interstitially around the middle of the neo-Y chromosome; the distance between the Rber248 and Rber299 was similar to the distance between the Rber299 and the end of the long arm. This situation occurred in 22.7% of the embryos (5 embryo) (Fig. 2b; Additional file 3). In pattern III Rber248 was located in the same position while Rber299 was interstitial, like in the pattern II, but closer to the Rber248, occurring in only one embryo (4.54% of the sample). The distance between the two satDNAs was closer than between Rber299 and the end of the long arm (Fig. 2c; Additional file 3). For the three variants I, II and III the neo-Y had a similar size as shown in the Fig. 3a. Variant IV occurred in only one individual (4.54%) and in this variant the neo-Y was larger than in I, II and III. Moreover the signals for the satDNAs Rber248 and Rber299 were positioned in the middle of the chromosome, but in inverse order in relation to the centromere than the other variants, i.e., the Rber299 was more proximal than the Rber248 (Fig. 2d).

For variant IV, we also mapped three other satDNAs repeats that were observed in the neo-Y variant I (the most common in mitosis), i.e., Rber1, Rber59 and Rber158. These three satDNAs were found arranged in distinct position on the neo-Y, and also differing in clusters number in comparison to variant I. The Rber1 and Rber59 were located in centromere, extending for the short arm, besides a proximal signal in the long arm. For Rber1 additional signal with interstitial position was also observed, similar to those observed in variant I (Fig. 3b). The Rber158, as observed in the variant I presented three discrete signals in interstitial position, but the band that occurred in the short arm of variant I was not noticed (Fig. 3b).

**Fig. 4:**
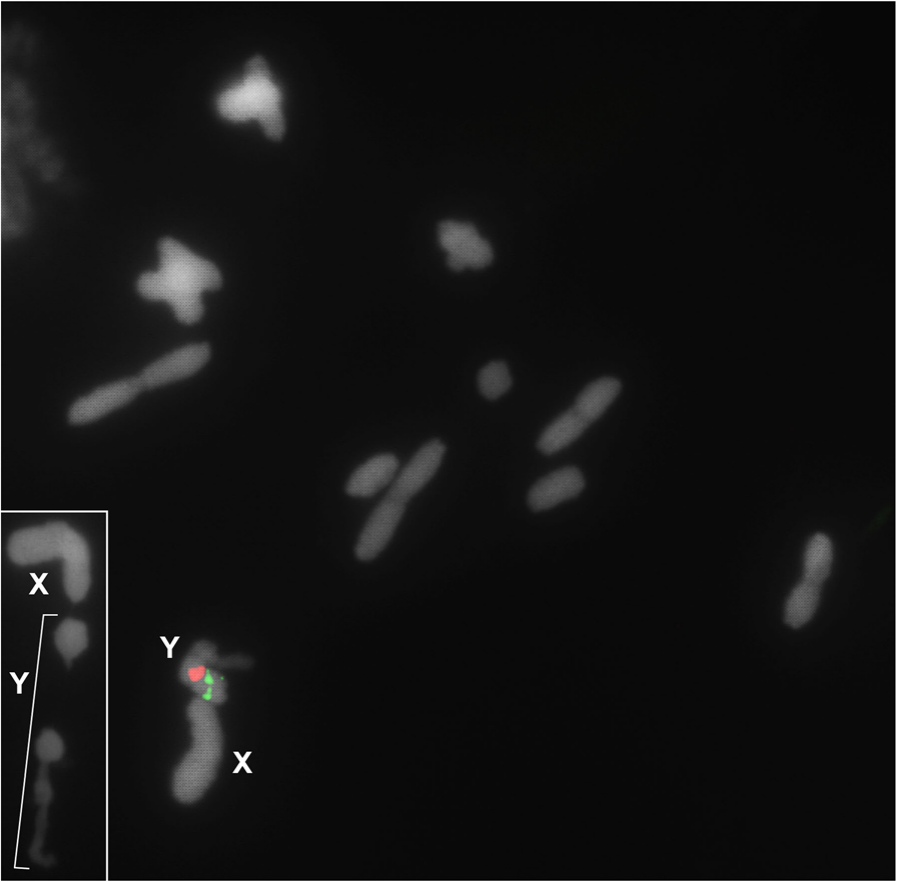
Male metaphase I probed for Rber248 and Rber299 showing the fragmented neo-Y chromosome. The inset shows the sex bivalent in early anaphase, highlighting the fragmentation of the neo-Y chromosome

In order to verify the neo-Y polymorphisms in the germline of adult males, we also performed analysis of meiotic cells at metaphase I. Among the four variants seen in embryos only three were observed in adults, i.e., I, II and III (Fig. 2a-c), and additionally another distinct variant (named variant V) not noticed in embryos was recognized, in which the Rber299 was placed terminally (Fig. 2e). Frequencies for the variants were different in comparison to those observed in embryos, i.e., variant I 7.15% (1 individual), variant II 57.1% (8 individuals), variant III 28.6% (4 individuals) and variant V 7.15% (1 individual). Additionally, in meiosis we found a neo-Y chromosome that was not possible to include in any of the described variants. This neo-Y was folding on itself at metaphase I, fragmented but joined by a thin chromatin in early anaphase I. The meiotic signals for Rber248 and Rber299 remained in the same position as those observed for mitotic variant I, II, II and V (Fig. 4).

## Discussion

### General organization of satDNAs in *R. bergii* genome

In grasshoppers RepeatExplorer satDNAs analysis was successfully applied in three species, revealing clues about organization of satDNAs in chromosomes of *Schistocerca gregaria* [52], general patterns of evolution of satDNAs using in *Locusta migratoria* [45] and the origin of the B chromosome in *Eumigus monticola* [46]. In comparison to the other species, the *R. bergii* satDNAs revealed differences concerning number of satDNA loci, clusterization for distinct families and specific chromosomal distribution, e.g., clustered in *R. bergii* and *S. gregaria*; clustered, dispersed and intermingled in *E. monticola* and *L. migratoria*, besides absence of band-like pattern detected through FISH [45, 46, 52]. In *R. bergii*, the absence of one satDNA occupying all centromeric or proximal areas of centromeres is remarkable, highlighting the differential centromere composition between chromosomes. In *S. gregaria* [52], *L. migratoria* [45] and *E. monticola* [46] at least one satDNA repeat was placed near to centromere and for *E. monticola* the pericentromeric location of EmoSat08-41 suggested involvement in centromeric function [46]. Our data suggest that if the satDNAs of *R. bergii* play a centromeric function as in other organisms [53–55], distinct centromeres are governed by distinct satDNA repeats or a combination of them. On the other hand, it is possible that these satellites might not play a centromeric function.

A noticed similarity between the three grasshopper species with satDNAs studied until now is the predominance of satDNAs families rich in A + T base pairs, suggesting that heavy satDNAs could be a common feature of grasshopper genomes. Considering that species studied belongs to distantly phylogenetically related families or subfamilies the sharing of A + T-rich satDNAs could be an ancient, which was conserved after satDNA library divergence. This picture is completely different from that observed in the cricket *Eneoptera surinamensis* in which the analysis of satDNAs revealed predominance of G + C-rich satDNAs [56], suggesting a more complex history for satDNA library in the ancestor of Ensifera and Celifera orthopterans, which diverged about 300 mya [57]. The most common A + T content of the *R. bergii* satDNAs is also a common hallmark for satDNAs embedded in the centromeric heterochromatin [53, 58]. A + T-rich centromeric satDNAs have been reported in other insect, such as *Tribolium castaenum* [59], *T. brevicornis* [60] and *Drosophila* species [53].

Our analysis revealed the occurrence of ten satDNAs that represent only about 1.072% of *R. bergii* genome. Even though grasshoppers present large genomes, the satDNAs represent a small fraction of their repetitive DNA content, e.g., *L. migrtoria* 2.39 and 2.74% in Southern and Northern lineages, respectively [45] and *E. monticola* about 1.79 and 1.91% in 0B and +B genomes, respectively [46]. An exception is *S. gregaria* where the characterized satDNAs correspond to about 30.4% of the genome content [52]. High amount of satDNAs were also reported in other insects, like the TCAST repeat that makes up 35% of the centromeric heterochromatin in *Tribolium castaneum* genome [61, 62]. These data suggest that in grasshoppers other repetitive elements could be more relevant for genome size increasing, like the Transposable Elements (TEs), as documented in *L. migratoria* with 60%, being ~24% and ~17% represented by DNA transposons and LINE retroelements, respectively [63].

*Ronderosia bergii* satDNAs lacks completely homology of satDNAs libraries from other grasshoppers, suggesting that satDNAs evolved repeatedly and independently diverging in each genome. Based on the divergence between satDNAs of *L. migratoria* and *E. monticola* Ruiz-Ruano et al. [46] proposed that in grasshoppers the satDNA lifespan is shorter than 100 mya, which according to Song et al. [57] corresponds to the most recent common ancestor for the two families to which these species belong, i.e. Acrididae and Pampharidae, respectively. Our data support this hypothesis considering that the diverging time between the Acrididae subfamilies Melanoplinae (*R. bergii*) and Oedipodinae (*L. migratoria*) is shorter than 100 mya [57].

### SatDNAs content highlights cryptic neo-Y variants and illuminates the complex evolutionary history of the *R.bergii* neo-sex chromosomes

The mapping of satDNAs and their distribution in the neo-XY bring new information about sex-chromosome composition and evolution in *R. bergii*. In a previous paper Palacios-Gimenez et al. [20] described the occurrence of distinct repetitive DNAs in the *R. bergii* sex chromosomes, like microsatellite and multigene families, highlighting the accumulation of repetitive DNAs and for some of them distinct accumulation between the neo-X and neo-Y, also distinct heterochromatinization. Here it is documented that the satDNAs are also composing the neo-XY, and could influence their evolution and differentiation through amplification for the ten distinct families mapped. Our findings confirm our previous hypothesis that the neo-sex chromosomes are highly divergent in *R. bergii*, where homologous sequences are completely loss between them, at least with the mapped repetitive DNAs. This differential accumulation could be documented, for example, by Rber59, Rber61, Rber370, Rber491, Rber520 sequences that are accumulated in the centromere of neo-X, but they are absent in the neo-Y; as well as by accumulation of Rber158 in neo-Y and the exclusive observation of Rber248 and Rber299 in the neo-Y. Differential patterns concerning the accumulation of repetitive DNAs and heterochromatinization are common hallmark of many sex chromosome systems with suppressed recombination [9, 64–68], and were reported in several well studied insect species, such as in the Y of *Drosophila melanogaster* [69] and *D. miranda* [66] and in the W chromosomes of many lepidopteran species (e.g. [70]).

The mapping of the two neo-Y specific satDNAs, i.e., Rber248 and Rber299, revealed the occurrence of five cryptic variants for this chromosome, caused most parsimonially by paracentric inversions after neo-XY establishment and after pericentric inversion established in the neo-Y. In animals, it is evident that chromosomal inversions trigger recombination suppression because of the lack of homologous sequences in those regions involved in the rearrangement [22–25]. The establishment of inversions is typically associated with recombination suppression and has been detected in other species with Y chromosome, such as in mammals [23], the three-spine stickleback fish [26], and the plant *Silene latifolia* [11], among others.

**Fig. 5:**
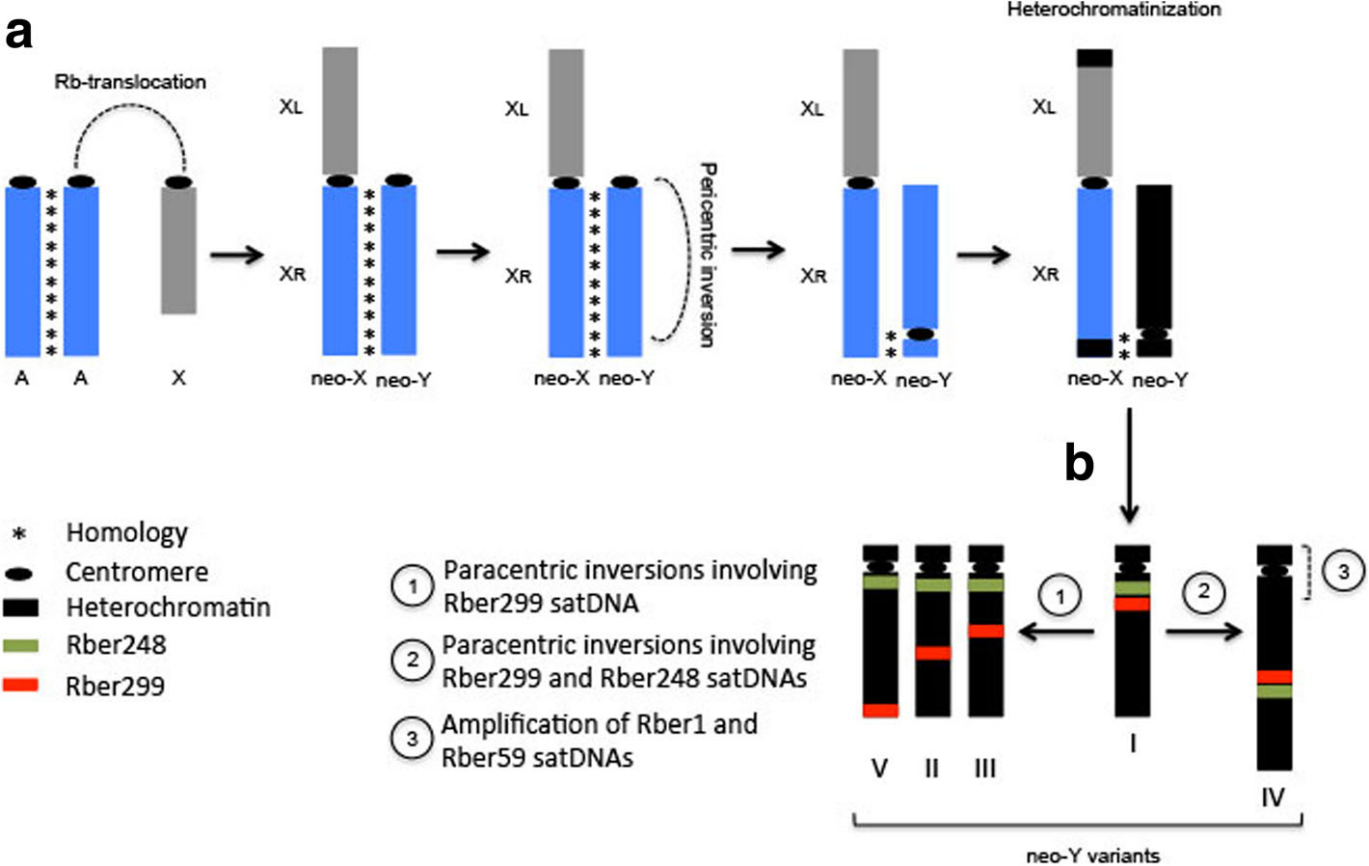
Hypothesis on the *R. bergii* neo-XY sex chromosome evolution based on satDNAs distribution patterns. **a** First the de novo origin of the neo-sex chromosomes involved an X-A centric fusion. After the neo-XY arisen, the neo-X and neo-Y presents conserved homology (indicated in blue) and recombining events are still able to occur between the XR arm and the neo-Y. A pericentric inversion has become established on the neo-Y involving more than 90% of its length followed by heterochromatinization (indicated in black) and recombination suppression between neo-XY. These rearrangements and recombination suppression could favored the accumulation of the satDNAs on the neo-Y and generate the different neo-Y variants as indicated in (**b**), as follows: (i) three large and independent paracentric inversions involving Rber299 displacing this satDNAs towards interstitial or distal regions originating the variants II, III and V; (ii) a paracentric inversion involving both Rber248 and Rber299 and (iii) amplifications of Rber1 and Rber59 (not observed in the variant I, probably by low copy number and FISH resolution) generating the pattern IV, in which the neo-Y is enlarged. Each satDNAs mapped on the neo-Y chromosome are indicated by colors directly on the images

Taken together, the former considerations and presented data lead us to suggest a model of evolution for the *R. bergii* neo-XY based on the satDNAs distribution patterns (Fig. 5). First the de novo origin of the neo-sex chromosomes involved an X-A centric fusion [27, 28] that is the most accepted hypothesis for the neo-sex chromosome origin in Orthoptera [15–17]. After the neo-XY arisen, recombining events are still able to occur between the XR arm, which corresponds to the ancestral autosome involved in the rearrangement, and the neo-Y, as documented in other Melanoplinae species by chiasmata analysis [17, 71]. After the neo-sex chromosomes have been established the C-shape disposition of neo-XY in meiotic metaphase led some authors to suggest the occurrence of a large pericentric inversion [15, 16] involving more than 90% of the neo-Y followed by its heterochromatinization and recombination suppression between the neo-X and the neo-Y [15, 16, 20]. These rearrangements and recombination suppression could have favored the accumulation of the satDNAs in the neo-Y, like Rber248 and Rber299 clustered near each other in the proximal region of long arm, nearby the centromere, as well as Rber1 in proximal position and Rber158 forming multiple clusters along the neo-Y. These characteristics were observed in the variant named I and specifically the Rber248 was closer to the centromere than Rber299. Afterward, it is more parsimonious from the variant I the occurrence of sets of chromosomal rearrangements generating the other variants, as follows: (i) three large and independent paracentric inversions involving Rber299 displacing this satDNAs towards interstitial or distal regions originating the variants II, III and V; (ii) a paracentric inversion involving both Rber248 and Rber299 and (iii) amplifications of Rber1 and Rber59 (not observed in the variant I, probably by low copy number and FISH resolution) generating the pattern IV, in which the neo-Y is enlarged. Our hypothesis as well as the occurrence of paracentric inversions are supported by the satDNA distribution observed in both the mitotic and meiotic cells (see bellow).

Some remarkable differences regarding the occurrence and frequency of variants were noticed between embryos and meiotic cells from adult males. For example, the variant V was observed exclusively in adults, while the variant IV was seen in embryos. In addition, a variant, that not fall in the other five ones was recognizable in meiosis. This neo-Y is folding itself at metaphase I and fragmented but joined by a thin chromatin at anaphase I. We suggested that this variant could be the result of multiple breakages involved in the inversions. Even though the neo-Y is largely non-recombining with the neo-X, this meiotic configuration could facilitate the propagation of tandem repeats by intra-chromosomal crossing over, which is of the mechanisms proposed for the propagation of tandem repeats along the genome [9, 72, 73], but it deserves more investigation.

The differences between frequency for the five variants observed in *R. bergii* embryos and adults, mainly for variants I and II, could be directly implicated with the evolutionary patterns at populational level involving selection and genetic drift, like proposed for other chromosomal rearrangments [25]*.* We noticed that the variant I occur in 68.2 and 7.15% in mitosis and meiosis, respectively, while the variant II result in 22.7 and 57.1% in mitosis and meiosis, respectively. It is more parsimonious to explain these differences as results of genetic drift. The higher frequency of variant II in adults could be favored probably by selection; however, we are aware about this second explanation because we do not have enough evidences of selection acting on the neo-Y chromosome. In any case a larger sample and animals from other populations should be studied to confirm these statements. If inversions in *R. bergii* are evolving neutrally and variation in their frequency is only driven by genetic drift, it must be expected that similar proportion for the two variants would be maintained, both in embryos and adults. But our data suggests that variant II is more favored, being more frequent in adults. Other populations should be investigated to test if this pattern is a local adaptation or different geographical niches support different neo-Y variants. The other three variants should be less favored than the variant II, occurring in lower frequencies in adults.

## Conclusions

Taken together, our results show accumulation and repositioning of satDNAs arrays resulting in an interesting cryptic polymorphisms for the *R. bergii* neo-Y. This suggests a complex evolutionary history of the neo-sex chromosomes than previously thought (see [20]), shedding light on understanding patterns of sex chromosome evolution in Orthoptera. There is no other case described until now in Orthoptera in which an established neo-Y chromosome is highly variable as in *R. bergii*. Because neo-sex chromosomes like those present in *R. bergii* could be an agent of species-level selection further analysis must be performed. Another interesting issue that allows to address these findings is at populational level. A large numbers of traits have been associated with inversion polymorphism in several taxa [74]. *Ronderosia bergii* is characterized by the highly variable patterns of body colour along its distribution range and in some cases within the same locality [29]. Until now, we do not know if in other distant populations of *R. bergii*, another neo-XY variant is favored by natural selection, and the frequencies found in meiosis and mitosis change following a polymorphism. If this occurs, it would be an interesting fact that could contribute to the knowledge of complex polymorphic populations presenting both the chromatic and morphological variants. Also, the neo-sex chromosomes may be involved in the evolution of complex population variants of *R. bergii*, which, in some cases, were erroneously described as different species.

FISH mapping using these markers in different populations, which included this phenotypic variations, will help us to understand the actual state of polymorphisms, and its possible influence on population isolation. In addition, controlled crosses using male with known neo-Y variant and analysis of offspring will give the possibility to test if inversions are or not occurring de novo*.*

## Additional files

Additional file 1: satDNAs monomer consensus sequences. (DOC 63 kb)

Additional file 2: Table displaying the size ratio Y/X of the neo-Y variants in distinct mitotic metaphases. For measurement of sex chromosome sizes from ten metaphases it was used one individual for each variant. (DOCX 25 kb)

Additional file 3: Distance between markers regarding the size of the neo-Y chromosome. In mitosis for variant II we studied five individuals and for variant III one individual. In meiosis for variant II we studied seven individuals, for variant III five individuals and for variant V one individual. For each individual five cells were analyzed. (DOCX 36 kb)

## Abbreviations

2n: Diploid number
bp: Base pairs
DAPI: 4′, 6-Diamidino-2-phenylindole
FISH: Fluorescence in situ hybridization
PCR: Polymerase chain reaction
