# Supplementary material for: Uncovering the evolutionary history of neo-XY sex chromosomes in the grasshopper Ronderosia bergii (Orthoptera, Melanoplinae) through satellite DNA analysis
Source: BMC Evol Biol. 2018 Jan 8;18:2. doi: 10.1186/s12862-017-1113-x (PMC5767042; doi:10.1186/s12862-017-1113-x)
Supplement: Supplementary file 1 — satDNAs monomer consensus sequences. (DOC 63 kb) [file 12862_2017_1113_MOESM1_ESM.doc]

**Additional file 1**

**Rber1 satDNA consensus sequence 52 bp**

1 10 20 30 40 50

| | | | | |

GTCCTATGTGATCAAAAGTATCCAGACAACCTGACAAAAGGTTTCAGACA

CA

**Rber59 satDNA consensus sequence 22 bp**

1 10 20 30 40 50

| | | | | |

GGTGTCTGCCTGCCAGTTCGCA

**Rber61 satDNA consensus sequence 11 bp**

1 10 20 30 40 50

| | | | | |

ACATACTCTGT

**Rber158 satDNA consensus sequence 177 bp**

1 10 20 30 40 50

| | | | | |

ACGCAACATTTTCGCCATTTCTCGGATGCTGTATCTGGTAATCGGCTCGG

TAATCTGTGTTAATTCTTTTACACACATCTGCAGATGCTTCCAGCTACTG

GTGGACAAAGTGGGATGTGGCGATGACGAATAGTTCTTTTTTTATAGACC

TCGAAACTTATGCCCGGAACTGTGCTT

**Rber185 satDNA consensus sequence 22 bp**

1 10 20 30 40 50

| | | | | |

GGCTAGCATAGTAAAGCTAGCA

**Rber248 satDNA consensus sequence 165 bp**

1 10 20 30 40 50

| | | | | |

GGAGATTTATTTACCACAGTATAACGATACAACAATCCGATACGGCTACA

GATACAGACTGAAACATGGTGCGAGTCGGCAGGAAGGAGAGAACTCTGTG

TCAGTTATAGGATCTGGGTACATGCAGTCGTATAATCACGTACACAAATT

CCTATTATTAACAAA

**Rber299 satDNA consensus sequence 285 bp**

1 10 20 30 40 50

| | | | | |

CTCGCAACAGTCAGACTCATTGTGGAGTATGGGTATCGCTGATAATAACG

ATTTCGGCGGCCAAAAAGCCATCTGTAGACCCCTATTCACGTAATTTAAC

GTAACTTTCCATGTGTGTACGAGTGTATTATGTTTTCACGATTACTCACG

CCTATTTGGCTTGTCATTACTCGCTCTCAAAAGTAGAATTGAAACTTTGA

GTAACGCGCTCCTATCCTACCTTTGAAAGCCGAGTTCCATATTCGATAGC

ATTCGAAGGCGCCTCGTGCACGGACCCAGAGTGTC

**Rber370 satDNA consensus sequence 16 bp**

1 10 20 30 40 50

| | | | | |

CAGGGAATACAAGTCA

**Rber491 satDNA consensus sequences 121 bp**

1 10 20 30 40 50

| | | | | |

ATCTTATACAATATAACGAACTGTCTCAGTATTCTGAGAAACAATTTATT

CTGGTTATACTGCAGTAACCTCCTTTTTTGGGAGTTCAAGAATGTCTGCA

GACAAAATTAACAGTGCTGTG

**Rber520 satDNA consensus sequences 5 bp**

1 10 20 30 40 50

| | | | | |

GATGT
