# Supplementary material for: Uncovering the evolutionary history of neo-XY sex chromosomes in the grasshopper Ronderosia bergii (Orthoptera, Melanoplinae) through satellite DNA analysis
Source: BMC Evol Biol. 2018 Jan 8;18:2. doi: 10.1186/s12862-017-1113-x (PMC5767042; doi:10.1186/s12862-017-1113-x)
Supplement: Supplementary file 2 — Table displaying the size ratio Y/X of the neo-Y variants in distinct mitotic metaphases. For measurement of sex chromosome sizes from ten metaphases it was used one individual for each variant. (DOCX 25 kb) [file 12862_2017_1113_MOESM2_ESM.docx]

**Additional file 2**

Table displaying the size ratio Y/X of the neo-Y variants in distinct mitotic metaphases. For measurement of sex chromosome sizes from ten metaphases it was used one individual for each variant.

|  | **Ratio Y/X in ten distinct metaphases** | | | | | | | | | |  |
| --- | --- | --- | --- | --- | --- | --- | --- | --- | --- | --- | --- |
| **Y variant (mitosis)** | **1** | **2** | **3** | **4** | **5** | **6** | **7** | **8** | **9** | **10** | **Mean** |
| **I** | 0.5 | 0.57 | 0.53 | 0.59 | 0.54 | 0.58 | 0.57 | 0.61 | 0.56 | 0.59 | 0.56 |
| **II** | 0.44 | 0.67 | 0.51 | 0.48 | 0.51 | 0.52 | 0.52 | 0.56 | 0.45 | 0.53 | 0.52 |
| **III** | 0.58 | 0.63 | 0.59 | 0.56 | 0.62 | 0.6 | 0.6 | 0.64 | 0.65 | 0.58 | 0.6 |
| **IV** | 0.72 | 0.79 | 0.76 | 0.73 | 0.72 | 0.8 | 0.82 | 0.82 | 0.79 | 0.85 | 0.78 |
