# Supplementary material for: Uncovering the evolutionary history of neo-XY sex chromosomes in the grasshopper Ronderosia bergii (Orthoptera, Melanoplinae) through satellite DNA analysis
Source: BMC Evol Biol. 2018 Jan 8;18:2. doi: 10.1186/s12862-017-1113-x (PMC5767042; doi:10.1186/s12862-017-1113-x)
Supplement: Supplementary file 3 — Distance between markers regarding the size of the neo-Y chromosome. In mitosis for variant II we studied five individuals and for variant III one individual. In meiosis for variant II we studied seven individuals, for variant III five individuals and for variant V one individual. For each individual five cells were analyzed. (DOCX 36 kb) [file 12862_2017_1113_MOESM3_ESM.docx]

**Additional file 3**

Distance between markers regarding the size of the neo-Y chromosome. In mitosis for variant II we studied five individuals and for variant III one individual. In meiosis for variant II we studied seven individuals, for variant III five individuals and for variant V one individual. For each individual five cells were analyzed.

|  | **Ratio between distance of Rber248-Rber299/Y size** | | | | |  | **Ratio between distance of Rber299-Y long arm end/Y size** | | | | |  |
| --- | --- | --- | --- | --- | --- | --- | --- | --- | --- | --- | --- | --- |
| **neo-Y variant (mitosis)** | **1** | **2** | **3** | **4** | **5** | **Mean** | **1** | **2** | **3** | **4** | **5** | **Mean** |
| **II** | 0.33 | 0.34 | 0.37 | 0.45 | 0.41 | 0.38 | 0.39 | 0.36 | 0.42 | 0.34 | 0.37 | 0.38 |
|  | 0.38 | 0.39 | 0.33 | 0.35 | 0.41 | 0.37 | 0.4 | 0.38 | 0.33 | 0.36 | 0.36 | 0.37 |
|  | 0.32 | 0.31 | 0.32 | 0.30 | 0.31 | 0.32 | 0.43 | 0.38 | 0.45 | 0.41 | 0.39 | 0.41 |
|  | 0.4 | 0.37 | 0.33 | 0.41 | 0.37 | 0.38 | 0.32 | 0.35 | 0.39 | 0.34 | 0.37 | 0.35 |
|  | 0.32 | 0.36 | 0.34 | 0.36 | 0.31 | 0.34 | 0.49 | 0.44 | 0.43 | 0.4 | 0.45 | 0.44 |
| **III** | 0.27 | 0.28 | 0.28 | 0.22 | 0.22 | 0.25 | 0.48 | 0.51 | 0.42 | 0.49 | 0.48 | 0.48 |
| **neo-Y variant (meiosis)** | **1** | **2** | **3** | **4** | **5** | **Mean** | **1** | **2** | **3** | **4** | **5** | **Mean** |
| **II** | 0.39 | 0.44 | 0.40 | 0.29 | - | 0.38 | 0.41 | 0.36 | 0.42 | 0.46 | - | 0.41 |
|  | 0.46 | 0.33 | 0.4 | 0.34 | - | 0.38 | 0.34 | 0.37 | 0.36 | 0.38 | - | 0.36 |
|  | 0.4 | 0.38 | 0.34 | 0.35 | 0.4 | 0.37 | 0.37 | 0.4 | 0.38 | 0.37 | 0.38 | 0.38 |
|  | 0.44 | 0.39 | 0.35 | 0.38 | 0.38 | 0.39 | 0.38 | 0.37 | 0.40 | 0.36 | 0.37 | 0.37 |
|  | 0.39 | 0.36 | 0.44 | 0.34 | 0.41 | 0.38 | 0.39 | 0.38 | 0.3 | 0.36 | 0.36 | 0.36 |
|  | 0.34 | 0.34 | 0.35 | 0.35 | 0.4 | 0.36 | 0.39 | 0.43 | 0.36 | 0.4 | 0.43 | 0.4 |
|  | 0.39 | 0.39 | 0.31 | 0.36 | 0.32 | 0.35 | 0.39 | 0.43 | 0.45 | 0.41 | 0.46 | 0.43 |
| **III** | 0.3 | 0.25 | 0.26 | 0.22 | - | 0.26 | 0.52 | 0.55 | 0.54 | 0.49 | - | 0.52 |
|  | 0.42 | 0.23 | 0.27 | 0.27 | - | 0.3 | 0.5 | 0.5 | 0.5 | 0.55 | - | 0.51 |
|  | 0.25 | 0.27 | 0.29 | 0.31 | 0.26 | 0.28 | 0.46 | 0.46 | 0.48 | 0.5 | 0.5 | 0.48 |
|  | 0.28 | 0.23 | 0.38 | 0.28 | 0.33 | 0.3 | 0.49 | 0.51 | 0.48 | 0.52 | 0.56 | 0.51 |
|  | 0.34 | 0.32 | 0.38 | 0.3 | 0.32 | 0.33 | 0.47 | 0.48 | 0.42 | 0.44 | 0.44 | 0.45 |
| **V** | 0.54 | 0.59 | 0.54 | 0.51 | 0.53 | 0.54 | 0.24 | 0.18 | 0.21 | 0.25 | 0.21 | 0.22 |
